# The Relevance of the Predominant Clonal Evolution (PCE) Model for the Molecular Epidemiology and Subspecific Taxonomy of *Trypanosoma cruzi*

**DOI:** 10.3390/pathogens14050407

**Published:** 2025-04-24

**Authors:** Michel Tibayrenc

**Affiliations:** MIVEGEC, IRD, CNRS, University Montpellier, 34000 Montpellier, France; michel.tibayrenc@ird.fr

**Keywords:** parasite, fungus, bacterium, Chagas disease, strain typing, clonality, evolution, taxonomy, species concept

## Abstract

The predominant clonal evolution (PCE) model is often misunderstood. Contrary to common belief, it is not restricted to strict mitotic clonality. Instead, it encompasses processes such as selfing, strong homogamy, and various forms of parthenogenesis, as widely acknowledged by researchers studying clonality. Moreover, the PCE model does not claim that genetic recombination is entirely absent or devoid of epidemiological and evolutionary significance. In this review, I will explore the reciprocal relationship between PCE and molecular epidemiology (strain typing) and discuss the implications of PCE for revising the subspecific nomenclature of *Trypanosoma cruzi*.

## 1. Introduction

Infection with *Trypanosoma cruzi* remains a critical health problem in Latin America and is becoming increasingly concerning in countries outside the traditional Chagas disease region, due to imported cases. Effective Chagas disease control necessitates socioeconomic measures alongside ongoing efforts in basic research, including the molecular evolution of *T. cruzi* [1].

Since the early 1960s, the advent of molecular tools such as Multilocus* Enzyme Electrophoresis* (MLEE*) and DNA-based markers—such as Microsatellites*, Multilocus Sequence Typing* (MLST*) [2], Pulse-Field Gel Electrophoresis* (PFGE*), Restriction Fragment Length Polymorphism (RFLP*), Randomly Amplified Polymorphic DNA (RAPD*), and Amplified Fragment Length Polymorphism (AFLP*)—have paved the way for the emergence of molecular epidemiology. Today, this field relies on highly discriminative markers such as metabarcoding* [3], low-stringency single-primer polymerase chain reaction (LSSP-PCR) [4], single nucleotide polymorphisms* (SNPs*), and culture-free genome-wide locus sequence typing (GLST) [5]. These molecular tools enable the tracking of epidemiological pathways by tagging pathogen strains with genetic markers.

For a long time, molecular epidemiology was based on an empirical interpretation of data: strains appearing identical based on a given marker were assumed to be identical, similar strains were considered similar, and highly divergent strains were deemed very different—without further inference. The integration of population genetic* and phylogenetic* concepts into molecular epidemiology—first applied to *Trypanosoma cruzi* [6,7] and later to other pathogens [8]—provided a deeper understanding of the evolutionary significance of pathogen strains. This advancement greatly enhanced the resolution and accuracy of molecular epidemiological studies.

The PCE model remains widely misunderstood by many researchers. Since it is highly relevant not only to molecular epidemiology but also to the subspecific taxonomy of pathogens, a fresh, updated clarification is warranted. This article does not aim to provide an exhaustive review of *T. cruzi* genetic diversity, but rather, to clarify the PCE model and its predictive power regarding *T. cruzi* subspecific variability.

## 2. Why Is Clonality vs. Sexuality Relevant for Both Molecular Epidemiology and Taxonomy?

In this paper, sexuality is broadly defined as any genetic exchange between different cells, whereas clonality encompasses all cases in which daughter cells are genetically identical or very similar to parental cells.

Both molecular epidemiology and subspecific taxonomy require stable “relevant units of analysis” (RUAs) [9]. If sexuality is frequent and leads to panmixia*, such stability is lost, as genetic lineages undergo constant recombination and quickly dissolve into the common gene pool.

This is why the long-standing clonality/sexuality debate [6,10,11] is crucial not only for understanding the fundamental biology of pathogens but also for designing molecular epidemiological tools and refining taxonomic classifications. To clarify this debate, it is essential to establish clear definitions of the terms “clone” and “clonality”.

## 3. Clones and Clonality: Multiple Meanings

The primary task of science is to establish clear definitions for the concepts under study. These definitions may vary between authors and according to the purpose of a given study. A term can have multiple definitions, provided they are clear and coherent.

“Clone” is often understood in a cytological sense, referring to a set of daughter cells derived from a single progenitor cell through mitotic* division. This interpretation is commonly referred to as “strict clonality” [12,13] or “absolute clonality” [14]

In this article, as in many previous ones, “clone” and “clonality” are strictly defined in genetic terms. Clonality is confirmed in all cases where daughter cells are genetically identical or highly similar to the parental cell. This genetic definition of clonality is widely accepted among scientists studying clonal reproduction on clonal (parthenogenetic) vertebrates, including fish, amphibians, and reptiles (see, for example, [15]).

Genetic clonality thus encompasses not only “strict” (mitotic) clonality but also self-fertilization*, extreme homogamy* (inbreeding*), and various forms of parthenogenesis*. These processes all produce genetically identical clones and have similar impacts on pathogen population structures. This crucial point is often misunderstood by authors who adhere strictly to the definition of “strict” (mitotic) clonality.

## 4. Misconceptions Regarding the PCE Model Being “Challenged”

Since its initial formulation [6,7,8], the PCE model has undergone significant refinement and clarification [16]. However, it has been incorrectly criticized on the following points:

### 4.1. Total Absence of Genetic Recombination/Sexuality

A fundamental principle of the PCE model is that it does not claim genetic recombination or sexuality is completely absent. Rather, it asserts that such events are not frequent enough to disrupt the predominant clonal structure of the species under study. The mere detection of genetic exchange, whether experimentally or in natural populations [12,13,17,18,19,20,21,22,23], does not in itself challenge the PCE model. What matters is the actual impact of these exchanges on the species’ population structure (see below).

Successful experimental recombination (e.g., [24]) provides valuable insights into the basic biology of *T. cruzi*. However, it does not indicate the frequency or significance of such events in natural populations of this parasite.

### 4.2. Genetic Recombination Has No or Little Evolutionary/Epidemiological Significance

The PCE model does not claim that genetic recombination or sexuality lacks evolutionary or epidemiological importance, as is sometimes mistakenly argued [21,25,26,27,28,29]. On the contrary, it is likely to be highly significant.

### 4.3. Use of Inadequate Genetic Tools

The PCE model is not an artifact resulting from outdated genetic approaches with insufficient resolution [22,30,31,32] or an insufficient number of markers [33]. For a long time [34], it has relied on the analysis of multiple genetic markers and the most recent high-resolution genomic* tools.

### 4.4. Improper Sampling

It has been suggested that the apparent lack or rarity of genetic exchange is not due to inherent biological properties of the species under study but rather to trivial geographical and/or temporal separation (the “Wahlund effect*” [33,35]; (see also “the starving sex hypothesis”). However, this criticism does not hold, as all studies addressing the PCE model have carefully accounted for this potential bias through rigorous sampling at both the sympatric and allopatric levels, across micro- and macro-geographical scales.

### 4.5. Inappropriate Evolutionary Scale

Another hypothesis [27,32] proposes that the apparent PCE in *T. cruzi* is due to genetic recombination/sexuality being inhibited between the species’ main phylogenetic subdivisions (discrete typing units* [DTUs*] or near-clades*; see below) but occurring, to some extent, within each of them. While this hypothesis is testable, it has been challenged by microevolutionary analyses (see “the Russian doll* model” below).

## 5. Main Features of the PCE Model and the Case of *Trypanosoma cruzi*

After outlining what the PCE model is not, here are its main characteristics.

### 5.1. Mandatory Analysis of Multiple Genetic Loci

PCE results from long-term, widespread inhibition of sexuality, genetic exchange, and recombination*. Since genetic recombination involves the exchange of genetic material across two or more loci, identifying PCE necessarily requires analyzing at least two loci—ideally, as many as possible. This necessitates surveying multilocus genotypes (MLGs). In species undergoing PCE, these MLGs provide the most precise definition of the often vague and widely used term “strain”. The more loci that are included in a study, the greater its resolution power.

This requirement invalidates studies based solely on the mini-exon gene, which has been widely used in *T. cruzi* research [36]. Since this gene family represents the variability of only a single locus, it cannot demonstrate a lack of recombination. However, once PCE has been confirmed through appropriate analyses, this marker can be used to characterize *T. cruzi* MLGs and genetic subdivisions [37]. Additionally, because genetic exchange can only be detected through variable loci, adding monomorphic loci does not enhance the analytical resolution.

Fortunately, since the pioneering studies on the genetic variability of the Chagas disease agent (*T. cruzi*) [38], numerous variable genetic loci have been analyzed, providing extensive data for research on genetic recombination.

### 5.2. Widespread Propagation of Unchanged MLGs Across Space and Time

This is the most intuitive consequence of clonal evolution. Under clonality, daughter cells are genetically identical—or nearly identical—to the parental cell, replicating as “genetic photocopies”. When this process persists over evolutionary timescales, these unchanged MLGs can be detected across vast geographic and temporal ranges, at frequencies incompatible with panmictic expectations.

For example, in *T. cruzi*, the MLEE MLG 39 has been sampled in multiple locations across Bolivia, Chile, and Brazil, from human hosts and triatomine bugs, since the early 1980s [39]. It continues to be sampled today. Similarly, MLEE MLG 19 has been identified in triatomine vectors, wild mammals, and human hosts in Chile, Brazil, Venezuela, Colombia, and Bolivia. Additional cases of widely distributed MLEE MLGs are detailed in [39,40].

The ubiquity of these clonal multilocus genotypes cannot be explained by the Wahlund effect. If Wahlund’s effect were responsible, these overrepresented MLGs would be confined to restricted geographical areas.

At higher resolution and shorter evolutionary timescales, widespread microsatellite MLGs have been identified in Bolivian *T. cruzi* strains (Messenger et al., 2015 [41]). Similarly, widespread MLST MLGs have been sampled across vast geographical distances in Brazil [2].

It is important to note that such widespread MLGs should not be considered “true” clones but rather families of closely related clones, or “clonets” (see below).

### 5.3. Linkage Disequilibrium (LD)

LD is a statistical measure indicating that genotypes at different loci do not recombine randomly, contrary to what would be expected under panmixia. In a panmictic system, knowing the genotype at one locus provides no predictive information about genotypes at other loci. This state is known as linkage equilibrium. LD, by contrast, describes the opposite scenario: knowing the genotype at one locus allows for high-probability predictions about genotypes at other loci.

This principle underpins the powerful technique of indirect typing, where a single genetic marker can be used to infer entire MLGs and DTUs. For example, PCR typing [42]; AFLP-PCR and PCR-RFLP [43]; amplification of a single gene, such as *TcSC5D* [44]; mini-exon gene analysis [37]; and fluorescent fragment length barcoding [45] are techniques that could be used.

Strong LD was detected early in *T. cruzi*, leading to the hypothesis that MLEE MLGs behave as genetic clones [6].

A particularly revealing case of LD occurs between genetic markers subject to different selective pressures, as this suggests that LD extends across the entire genome of the species. In *T. cruzi*, LD has been demonstrated between MLEE and RAPD [46,47], as well as between MLEE, RAPD, and PCR-RFLP [48]. LD has also been observed between the polymorphism of 12 antigen loci on one side, and MLEE/RAPD on the other [49].

LD in *T. cruzi* is also detectable on microevolutionary scales (see below: Russian Doll Evolution).

### 5.4. Multigene Bifurcating Trees (MGBTs)

MGBTs serve as a proof-by-contradiction for PCE. If recombination was frequent, it would erase any durable phylogenetic signal over time. The persistence of MGBTs, as well as their close similarities across ecosystems (wild vs. domestic), hosts (triatomine bugs vs. mammals), and geographic regions, provides the strongest evidence for clonal evolution. This has been noted by multiple authors working on the population genetics of pathogens [50,51,52,53,54].

MGBTs are ubiquitous in *T. cruzi*, both at the species subdivision level (see below: discrete typing units and near-clades) and at lower evolutionary scales (see below: Russian Doll Evolution).

## 6. Additional Key Concepts Related to the PCE Model

### 6.1. Clonets

The so-called “clones” identified through a given set of genetic markers are not true clones but rather families of closely related clones. Using markers with higher resolution power will further subdivide these “clones” into smaller units. The term “clonet” refers to MLGs* defined by a specific set of genetic markers in species undergoing PCE [55].

**Figure 1:**
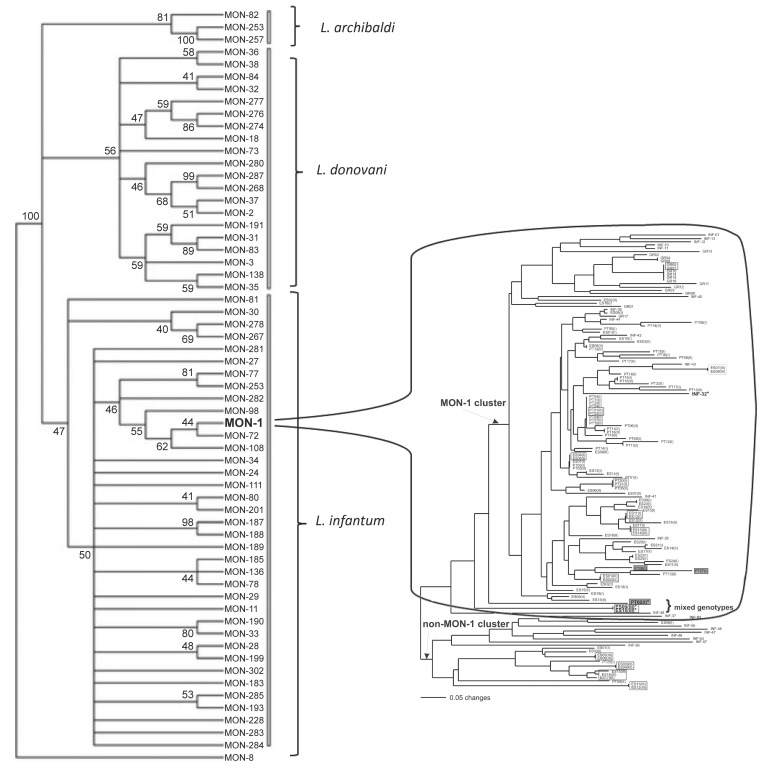
An illustration of the clonet concept and a typical Russian doll pattern* (RDP*) in *Leishmania infantum*. (**left**) A phylogenetic tree of the *Leishmania donovani/infantum* complex, based on MLEE analysis (adapted from Figure 1 in [56]). (**right**) The MLEE multilocus genotype “MON-1”, which represents only a small fraction of the *L. infantum* species (**left**), is revealed to be highly heterogeneous and structured when analyzed using microsatellites, which offer greater discriminatory power than MLEE (adapted from Figure 2 in [57]). MON-1 is a typical MLEE clonet: rather than a single clone, it constitutes a family of closely related clones.

For instance, MLEE MLGs are clonets characterized by MLEE analysis. The widespread MLEE MLGs 19 and 39 [39,40] are such clonets. If microsatellites are used—offering greater resolution—additional genetic variability within each of these clonets becomes evident (Figure 1).

### 6.2. Discrete Typing Units (DTUs)

DTUs, a term coined to define molecular epidemiology units for pathogen species (Tibayrenc, 1998), have been widely used for *T. cruzi*. This concept is purely descriptive, without inferring evolutionary significance, though DTUs have been proposed as equivalent to clades [58].

Six primary DTUs have been identified within *T. cruzi* via both MLEE and RAPD: DTUs 1, 2a, 2b, 2c, 2e, and 2f [47]. These were later renamed Tc I to VI [59]. A seventh DTU, specifically isolated from bats, was identified [60].

### 6.3. Near-Clades

PCE does not imply an absolute absence of recombination. Actually, it is highly probable that strictly clonal pathogens do not exist. However, in many pathogen species, distinct phylogenetic lineages are clearly present [16]. Occasional genetic exchange makes the term “clade” inappropriate to describe them, as a clade is a genetic lineage that is strictly isolated from others. Now, genetic isolation is not absolute among these near-clades. Moreover, the near-clades Tc V and VI appear to have a hybrid origin [61,62]. The term “clade” is thus unsuitable in this case, since a clade is expected to have a unique ancestor. However, the concept of near-clade remains valid for these hybrid lineages. The term “near-clade” [34] was specifically coined to designate pathogen phylogenetic lineages whose distinctness is somewhat blurred by occasional genetic exchange. Nevertheless, near-clades can be clearly identified through phylogenetic analysis and remain stable over space and time. Near-clades thus correspond to DTUs whose evolutionary origins have been clarified. The near-clade concept offers a valuable means to simplify the otherwise confusing terminology found in the literature on pathogen genetic diversity (Table 1).

### 6.4. Russian Doll Evolution; Russian Doll Patterns (RDPs)

In many pathogen species subdivided into major genetic units (DTUs; near-clades), the population structure and evolutionary traits observed within each near-clade resemble a miniature version of the overall species structure (Russian Doll Evolution [63]; Figure 1). This includes (i) widespread, stable MLGs; (ii) LD; and (iii) smaller near-clades identified by MGBTs. This indicates that PCE also operates within each near-clade at a microevolutionary level. This challenges the hypothesis [27,32] that posits that genetic isolation exists between the main genetic subdivisions (DTUs; near-clades) but is significantly weaker within them.

**Figure 2:**
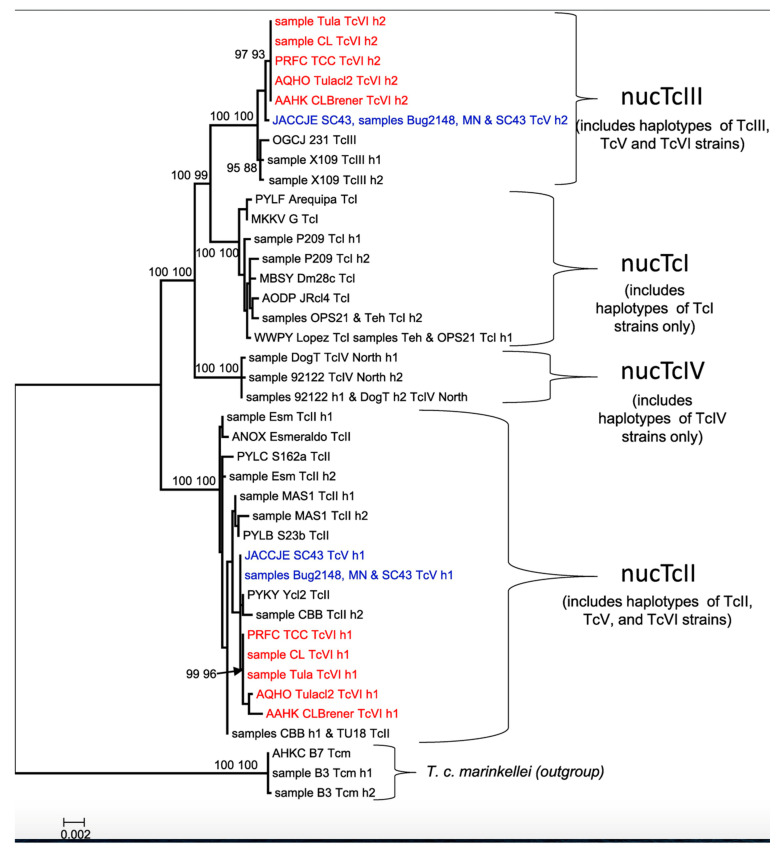
A concatenated phylogenetic tree of several nuclear genes, clearly showing smaller near-clades (RDPs) within each of the main near-clades of *Trypanosoma cruzi* [67].

RDPs are widespread in *T. cruzi* and are observed across all six major DTUs/near-clades of this parasite. In Ecuadorian TcI stocks, Costales et al. [64] identified highly significant LD, with this DTU/near-clade clustering into two smaller near-clades statistically associated with sylvatic and domestic cycles. RDP was also observed within TcI [65] using microsatellite analysis. The distribution of smaller near-clades suggests partial geographic separation, though this factor alone does not fully explain the results (Figure 1). The authors found statistically significant LD within all populations, concluding that they predominantly evolved clonally. Llewellyn et al. [66] also detected strong LD within the formerly designated DTU TcIIc (now TcIII). RDP is clearly demonstrated in a recent study [67] that analyzed a concatenated phylogenetic tree* involving multiple DNA sequences (Figure 2).

**Figure 3:**
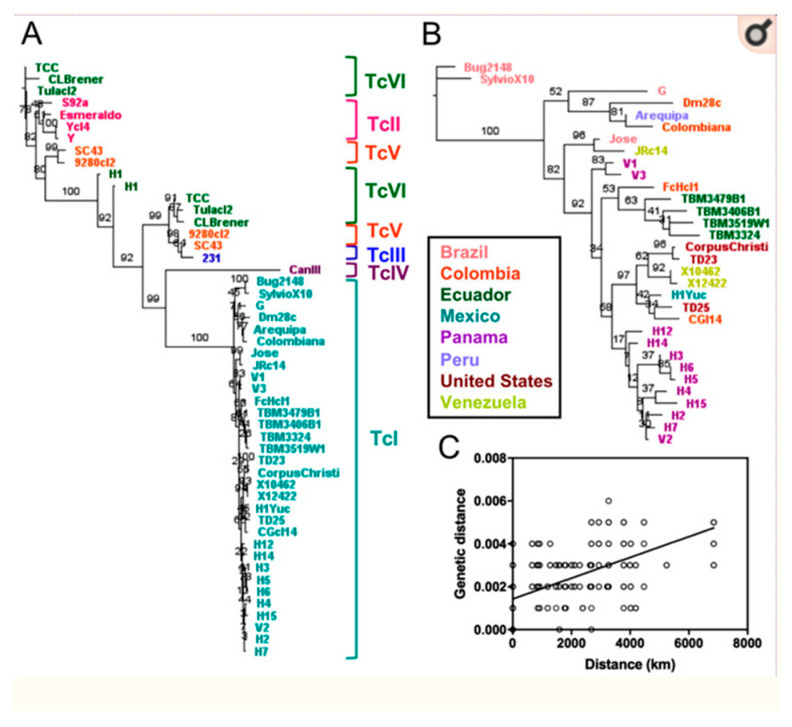
Two phylogenetic trees from concatenated sequences: (**A**) the whole *T. cruzi* taxon is subdivided into 6 near-clades or DTUs. (**B**) Additional subclustering within the TcI near-clade (Russian doll pattern). (**C**) Mantel test showing the correlation between geographical and genetic distances. (after Figure 5 of [68]).

Majeau et al. [68] constructed a concatenated phylogenetic tree based on the sequences of 30 nuclear genes. Their analysis confirmed a clear clustering into six main DTUs and revealed a strong RDP within TcI (Figure 3).

Genetic clustering within TcI is further illustrated in Supplementary Figure S1 of [69].

Regarding North American strains, Flores-López et al. [70] produced a concatenated tree based on four loci, revealing a clear RDP within TcI, including group III-V-VI, TcIV, and its variant TcIV “USA” (their Supplementary Figure S5).

Messenger et al. [41], in a comprehensive survey of Bolivian *Trypanosoma cruzi* strains involving 199 clones from 68 isolates and utilizing 26 microsatellite loci plus 10 mitochondrial maxicircle genes, revealed clear substructuring (smaller, related clades; RDP) within the TcI lineage. Not only were smaller near-clades detected, but highly significant LD levels were also found, even within the smallest near-clades, along with repeated MLGs at frequencies incompatible with panmixia. Substructuring was partly explained by geographic separation. However, this Wahlund effect alone does not fully account for the observed patterns. Furthermore, 65 out of 68 isolates were multiclonal, providing ample opportunities for mating (see below: “starving sex hypothesis”). While microsatellite (nuclear) and mitochondrial gene phylogenies were generally congruent, some discrepancies were noted, possibly due to genetic exchange. Alternative explanations include differing selective pressures and evolutionary rates, which are not mutually exclusive.

### 6.5. Is Apparent Clonality in T. cruzi Attributable to a Lack of Mating Opportunity? The “Starving Sex” Hypothesis

The “starving sex” hypothesis [71] can be considered a specific case of the Wahlund effect, positing that mating is restricted because different genotypes are not present simultaneously in the same host. More broadly, it encompasses all cases where physical barriers (spatial or temporal) are the only obstacles to genetic exchange.

This hypothesis was proposed by Cibulskis [72] to explain apparent clonality in *T. cruzi*. However, multiple instances of multiclonal infections have been recorded in this parasite, both in hosts [73] and triatomine vectors [39]. Flores-López et al. [70] also reported mixed infections of TcI and TcIV in the USA.

Additionally, various multiclonal infections have been observed at a microevolutionary level among microclones characterized by microsatellites [41]. The “starving sex” hypothesis is therefore not a parsimonious explanation for *T. cruzi* clonality, as this parasite has ample opportunities for mating in its natural cycles.

## 7. Conclusions and Future Direction

*T. cruzi* exhibits all the hallmarks of the PCE model, which in turn provides excellent predictive power regarding its population genetic structure. Without exception, no *T. cruzi* population has been found to be panmictic, meaning that random genetic exchange does not occur. Meiotic processes have been detected in TcI [74], which is a highly relevant discovery in terms of basic evolution. However, this discovery does not support the claim [75] that these populations are “almost panmictic”. These populations still display strong LD and smaller near-clades (Russian doll patterns) and thus conform to the PCE model. Meiosis is insufficiently frequent to disrupt the prevailing pattern of clonal evolution.

All surveyed *T. cruzi* populations exhibit widespread MLGs, LD, and MGBTs at all evolutionary scales.

From a molecular epidemiology perspective, clonets, DTUs, and near-clades provide practical analytical units for epidemiological surveillance due to their two key properties: (i) stability across space and time and (ii) ubiquity.

For the same reasons, DTUs/near-clades serve as a valuable framework for studying the medically relevant properties of the Chagas disease agent, including the epidemiology, pathogenicity, drug susceptibility, and vaccine potential. In particular, the major near-clades (TcI-VI) represent a promising resource for experimental evolution in *T. cruzi*, warranting further exploration.

Lastly, smaller DTUs/near-clades within TcI-VI could serve as the foundation for a subspecific taxonomy of *T. cruzi,* should experts deem it appropriate. This has already been proposed for the near-clade TcI [42,76]. However, it is advisable to adopt a flexible, informal nomenclature to avoid an excessive proliferation of Latin names.

The medical relevance of the PCE concept lies in its clear utility for molecular epidemiology (strain typing) and future research into the potential distinctive properties of the smaller near-clades (pathogenicity, treatment resistance).

Future development in this field should definitively rely on the latest progress in genomic studies, encompassing whole genome sequencing and Multi-SNP typing.

*T. cruzi* is not an isolated case; many pathogenic species, including bacteria, protozoa, fungi, and yeasts, exhibit similar PCE patterns, suggesting a convergent evolutionary trend among parasitic organisms [77].

## Figures and Tables

**Table 1:** The numerous imprecise terms used to describe genetic clusters within pathogen species. The near-clade concept provides a useful alternative to this confusing terminology. Clonets, DTUs, and near-clades serve as convenient and relevant units of analysis (RUAs) [9] for both basic and applied research.

Viruses	Bacteria	Parasitic Protozoa	Fungi
clades	clades	assemblages	AFLP groups
clusters	clonal complexes	clades	clades
genogroups	clonal lineages	clonal haplogroups	clonal groups
genotypes	clonal subgroups	clonal haplotypes	clonal lineages
groups	clusters	clonal lineages	clusters
lineages	eBurst groups	clonal types	clonal groups
major genotypes	family strains	clones	genetically distinct subgroups
major lineages	genetic groups	clonotypes	genotypes
phylogenetic groups	genoclouds	clusters	genotypic groups
phylogroups	genogroups	core subgroups	groups
subclades	genome groups	discrete typing units (DTUs)	lineages
subgenotypes	genomospecies	divergent entities	major clades
subgenotype clusters	genospecies	genetic clades	minor clades
subgroups	groups	genetic groups	molecular genotypes
sublineages	haplotypes	genetic types	molecular types
substrains	lineages	genotypes	phylogenetic species
subtypes	major branches	groups	subclades
subvariants	major clusters	haplogroups	subclusters
types	main/major lineages	haplotypes	subgenotypes
variants	major phylogenetic groups	lesser subgroups	subgroups
	phylogenetic clades	lineages	subpopulations
	phylogenetic groups	main haplogroups	varieties
	phylogenetic groupings	major clades	
	phylogroups	major clonal lineages	
	populations	major groups	
	primary clusters	major monophyletic groups	
	principal genetic groups	phylogenetic lineages	
	pulsotypes	populations	
	secondary clusters	subassemblages	
	semi discrete lineages	subclades	
	subclades	subclusters	
	subclones	subgroups	
	subclusters	sublineages	
	subgroups	subpopulations	
	sublineages	subgenotypes	
	subpopulations	subgroups	
	subspecies	subspecies	
	subspecies groups	subtypes	
	subtypes	subtype groups	
		types	

## Data Availability

Data are contained within the article.

## Glossary of Specialized Terms

Amplification fragment length polymorphism (AFLP)	Selective amplification of genomic restriction fragments (obtained by RFLP) by PCR*.
Barcoding	The DNA barcode. Some DNA fragments are highly conserved within the same species and variable between species. These are genetic markers or barcodes. cf. metabarcoding.
Clade	An evolutionary lineage defined by cladistic analysis. A clade is monophyletic (it has a single common ancestor) and genetically isolated (it evolves independently) from other clades, with no genetic exchange. See cladistic analysis.
Cladistic analysis	A method of phylogenetic analysis that relies on the polarization of characters, distinguishing between ancestral (plesiomorphic) and derived (apomorphic) traits. Only apomorphic characters shared by all members of a given clade (synapomorphic characters) are considered phylogenetically informative.
Concatenated phylogenetic tree	Method of constructing a phylogenetic tree by concatenating different gene sequences that have been aligned into a supergene matrix.
Discrete typing unit (DTU)	A group of genetic stocks that are more closely related to each other than to any other stock, remain stable over time and space, and can be identified by specific genetic, molecular, or immunological markers called tags. DTUs serve as reliable analytical units in studies considering pathogen genetic diversity and are ideal targets for molecular epidemiology surveys.
Genetic recombination	The exchange of genetic material between different individuals at two or more genetic loci, resulting in offspring with genetic combinations distinct from those of either parent.
Genomics	While genetics focuses on the study of individual genes, genomics examines the entire genome as a whole.
Homogamy	The tendency of an organism to mate with individuals that are genetically very similar or identical to itself.
Inbreeding	cf. homogamy.
Isoenzymes	Different electrophoretic variants of a given enzyme reflecting the genetic variability of the gene encoding that enzyme within the surveyed population. Variations in migration patterns among isoenzymes of the same enzyme arise from differences in their overall electric charge, which is determined by the specific electric charges of the amino acids composing the enzyme. Consequently, electrophoretic differences correspond to variations in the amino acid sequence and, ultimately, differences in the upstream gene sequence.
Linkage disequilibrium	The nonrandom association of genotypes at different genetic loci. In a population with no linkage disequilibrium (i.e., random genetic recombination), knowing an individual’s genotype at one locus does not provide information about their genotype at another locus. For example, in a randomly mating human population, knowing an individual’s ABO blood group does not indicate their Rhesus blood group. Conversely, if such an association is observed, it suggests linkage disequilibrium, indicating limited genetic recombination, which can be quantified using various statistical methods.
Locus	The physical location of a gene on a chromosome. By extension, in genetic terminology, the term locus may also refer to the gene itself (plural: loci).
Metabarcoding	Use of Next Generation Sequencing. Metabarcoding is an extension of barcoding through the use of NGS technology, which allows blind and one-time identification of all species present in a sample. cf. barcoding.
Microsatellite	A short DNA sequence, typically 1–4 base pairs long, repeated in tandem along the DNA molecule. In many species, including pathogens, the number of repeats varies significantly between individuals and across populations and pathogen strains. The number of repeats at a specific locus defines microsatellite alleles. Microsatellites are present at hundreds of locations in most species. These markers evolve rapidly and offer high-resolution analysis.
Mitosis	Equational cell division that gives rise to genetically identical cells.
Multilocus	Referring to a trait involving multiple loci.
Multilocus enzyme electrophoresis (MLEE)	Isoenzyme* analysis involving a wide range of enzyme systems, each corresponding to one or more genetic loci. Multilocus enzyme electrophoresis (MLEE) has been extensively applied in population genetics across a diverse array of living organisms, including pathogens.
Multilocus genotype	A combined genotype determined by multiple genetic loci.
Multilocus sequence typing (MLST)	A method for characterizing pathogens based on sequencing several housekeeping genes.
Panmixia: panmictic, panmictic expectations	A genetic structure in which genetic exchange occurs randomly within a given population. Panmictic expectations are the confirmation of this state through various population genetics tests.
Parthenogenesis	A mode of reproduction observed in certain metazoans (e.g., insects, amphibians, fish, reptiles) that occurs without the genetic contribution of a mating partner.
Phylogenetics	A branch of genetics dedicated to reconstructing the evolutionary history and relationships of taxa or distinct evolutionary lineages.
Polymerase chain reaction (PCR)	A technique that amplifies the complementary strands of a target DNA sequence through a series of cycles until the desired amount of DNA is produced. PCR employs synthesized primers whose nucleotide sequences are complementary to the DNA flanking the target region. The DNA is heated to denature and separate the complementary strands and then cooled to allow the primers to bind to the flanking sequences. Taq DNA polymerase is added, and the reaction undergoes the necessary number of replication cycles to achieve amplification.
Population genetics	The study of genetic variation across space and time within and among populations. This field emphasizes the population or species as a whole rather than individual organisms (see also population genomics).
Population genomics	The study of genomic variation across space and time within and among populations.
Pulse field gel electrophoresis (PFGE)	The separation of large DNA fragments achieved through a specialized electrophoresis technique that uses alternately pulsed, perpendicularly oriented electrical fields. Strains sharing the same pulsed-field gel electrophoresis (PFGE) pattern are referred to as pulse types. In bacteria, the large DNA fragments are produced by the action of a low-frequency restriction enzyme (a bacterial endonuclease that cuts at a low frequency) on the bacterial chromosome. As such, PFGE is a specific form of restriction fragment length polymorphism (RFLP). In the case of parasitic protozoa (such as *Trypanosoma* and *Leishmania*) and yeasts, the large DNA fragments represent entire chromosomes, reflecting the organism’s molecular karyotype.
Random primer amplified polymorphic DNA (RAPD)	In the classical polymerase chain reaction (PCR) method, the primers used are known DNA sequences, whereas the RAPD (random amplified polymorphic DNA) technique relies on primers with arbitrarily determined sequences. RAPD primers are typically 10 base pairs long, and the possible combinations are virtually unlimited. For a given genotype of an individual or strain, different primers will reveal different polymorphisms. RAPDs are an extremely powerful method for exploring the genetic variability of organisms. However, their use in routine strain identification is limited due to their lack of reproducibility.
Recombination (genetic)	cf. Genetic recombination.
Restriction fragment length polymorphism (RFLP)	DNA variability in a given organism can be detected using bacterial restriction endonucleases. These enzymes cut DNA at specific restriction sites defined by particular DNA sequences. The resulting polymorphism in DNA fragments can be visualized on gels, either directly through ethidium bromide staining or via Southern blot hybridization using specific probes.
Selfing: self-fertilization	Fertilization of an organism by itself, hence by a genotype that is identical to itself.
Sexuality	In this context, sexuality is used broadly to encompass all forms of genetic exchange between two distinct cells or individuals.
Single nucleotide polymorphism	Polymorphisms resulting from single nucleotide variations in the DNA sequence, known as single nucleotide polymorphisms (SNPs), contributing to differences among individuals, populations, and pathogen strains. SNPs are commonly used as high-resolution population markers.
Wahlund effect	In population genetics, the Wahlund effect traditionally refers to a heterozygote deficiency arising when two genetically distinct populations with different allele frequencies are mistakenly considered a single population, despite being separated by physical barriers (e.g., time or space). Here, we extend the term to refer to any apparent deviation from panmixia that results solely from physical obstacles (e.g., time or space) impeding genetic exchange.
